# Amelioration of cardio-renal injury with aging in dahl salt-sensitive rats by H_2_-enriched electrolyzed water

**DOI:** 10.1186/2045-9912-3-26

**Published:** 2013-12-02

**Authors:** Wan-Jun Zhu, Masaaki Nakayama, Takefumi Mori, Kiyotaka Hao, Hiroyuki Terawaki, Junichiro Katoh, Shigeru Kabayama, Sadayoshi Ito

**Affiliations:** 1 Center for Advanced and Integrated Renal Science, Tohoku University, Sendai, Japan; 2 Medical Device, Nihon Trim Co., Ltd, Osaka, Japan; 3 Department of Kidney and Hypertension, Fukushima Medical University, Fukushima, Japan; 4 Department of Cardiovascular Medicine, Tohoku University Graduate School of Medicine and Tohoku University Hospital, Sendai, Japan

**Keywords:** Aging, Cardiac remodeling, Chronic kidney disease, Hydrogen molecule, Electrolyzed water

## Abstract

**Abstract:**

Recent studies have revealed the biological effects of H_2_ in suppressing organ injuries due to acute inflammation and oxidative stress. Dahl salt-sensitive (SS) rats naturally develop elevated blood pressure (BP) and kidney injury with aging. The present study examined the effect of long-term supplementation of H_2_ in drinking water on age-related changes.

Four-week-old male Dahl SS rats were fed 3 types of water (n = 30 each) for up to 48 weeks: filtered water (FW), water with a high H_2_ content (492.5 ppb) obtained with water electrolysis (EW), or dehydrogenated EW (DW). Animals were subjected to histological analysis at 16, 24, and 48 weeks.

The FW group showed progressive BP elevation and increases in albuminuria and cardiac remodeling during the course of treatment. Histologically, there were significant changes as a function of aging, i.e., glomerular sclerosis with tubulointerstitial fibrosis in the kidney, and increased cardiomyocyte diameter with interstitial fibrosis in the heart at 48 weeks. These changes were related to the enhanced inflammation and oxidative stress in the respective organs. However, there were no striking differences in BP among the groups, despite histological alterations in the EW group being significantly decreased when compared to FW and DW in both organs, with concurrently lower oxidative stress and inflammatory markers at 48 weeks.

**Conclusion:**

Long-term *ad libitum* consumption of H_2_-enriched electrolyzed water can ameliorate the processes of kidney injury and cardiac remodeling with aging in Dahl SS rats by suppressing, at least partly, elevated inflammation and oxidative stress.

## Introduction

Dahl salt-sensitive (SS) rats develop blood pressure (BP) elevation accompanying kidney injury and cardiac remodeling on a high-salt diet [1-5]; however, anti-oxidants, such as ascorbic acid, tocopherol, and tempol, have been shown to be effective in ameliorating these changes [2-19]. Interestingly, it is known that Dahl SS rats, even on a normal salt diet, naturally develop BP elevation and kidney injury with aging [8].

Electrolysis gives rise to unique properties in water at the cathode side, such as alkalinity, low dissolved oxygen, and high dissolved hydrogen (H_2_) [14]. It has been reported that H_2_ water can suppress the generation of superoxide anions and hydrogen peroxide during the oxidative process [1], and decrease oxidative injury to DNA *in vitro*[6-14].

Recently, the biological action of H_2_ as a novel anti-oxidant has been an issue of focus [12]. Numerous animal studies have shown that administration of water-dissolved H_2_ suppresses acute ischemic and inflammatory injuries to various organs, such as the brain [10-15], liver [3], intestine [1], heart [20], and lung [7]. We previously reported the effect of *ad libitum* drinking of electrolyzed water (EW) on suppressing cardiac inflammation and fibrosis induced by acute ischemic reperfusion of unilateral kidneys in Dahl SS rats [20].

The present study examined the effect of long-term drinking of H_2_-enriched EW on age-related changes in BP, as well as kidney and heart injuries in Dahl SS rats fed a normal salt diet.

Herein, we show that long-term *ad libitum* consumption of EW ameliorated age-related cardio-renal injury in this model. This study supports the potential of utilizing H_2_ water as a novel anti-aging strategy.

## Methods

### Animals and protocols

Four-week-old male Dahl SS rats (n = 90) were housed in a temperature- and humidity-controlled room with 12-h light/dark cycles and provided a normal diet (0.5% NaCl). Three types of drinking water were prepared: filtered water (FW); water with a high content of dissolved H_2_ created by water electrolysis (EW); and dehydrogenated EW (DW). At the age of 5 weeks, the rats were divided into three groups of 30 subjects each for *ad libitum* drinking of the respective water types. Fresh water samples were produced twice a day, and delivered by a metallic straw from a closed bottle. EW was generated using a TRIM ION TI-9000 system (Nihon Trim, Osaka, Japan) [3,10]. The properties of the EW are shown in Table 1.

**Table 1:** Properties of the three water types used in the study

**Water type**	**pH**	**Dissolved hydrogen (ppb)**	**Redox potential (mV)**
FW	8.3 ± 0.0	1.6 ± 0.6	+140.1 ± 4.2
EW	10.4 ± 0.0	492.5 ± 17.8	−148.0 ± 4.0
DW	10.1 ± 0.2	3.0 ± 1.7	+85.8 ± 5.0

(mean±*SEM*).

*FW* filtered water, *EW* water with dissolved H_2_ produced by electrolysis, *DW* dehydrogenated EW.

During the course of the study, daily measurements were made of the volume of drinking water consumed. BP was measured every other week in the morning using the tail-cuff method. Urinary and blood samples were collected at 16, 24 and 48 weeks, and were measured using an auto-analyzer [20].

Whole kidneys and hearts were collected for histological examination and blood samples were collected from the aorta. Three experimental endpoints were employed: 16 weeks, 24 weeks, and 48 weeks; 10 rats per group were sacrificed at each time point. The rats were anesthetized using 1% pentobarbital (0.20 mg/kg) administered intraperitoneally. All procedures were performed in accordance with the institutional guidelines for the care and use of laboratory animals, and all protocols were approved by the Animal Committee at Tohoku University School of Medicine.

### Histological examinations

Kidney and heart samples were subjected to histological examinations after staining, using the Elastica-Masson method for determining renal injury and cardiac fibrosis, with the following parameters assessed: glomerular adhesions, glomerular sclerosis index (GSI), renal fibrosis, cardiac fibrosis, and cardiomyocyte diameter using Image J software (National Institute of Health (NIH), Bethesda, MD, USA) as reported elsewhere [3]. Immunohistochemical analysis was performed using monoclonal antibodies against ED-1 (Serotec, Oxford, UK), malondialdehyde (MDA) (JaICA, Shizuoka, Japan), nuclear factor erythroid 2-related factor (Nrf2) (Abcam, Cambridge, UK), and nitrotyrosine (NT) (Temecula, CA, USA). Slides were incubated overnight at 4°C with the primary antibodies, followed by analyses of ED-1 and NT [20]. For assessing MDA staining, five areas of the kidney or heart were randomly selected and the percentage of MDA-positive areas in each was measured by Image J software. For Nrf2 quantitation, the number of positive cells in heart tissue was counted in five randomly selected images.

### RNA preparation and quantitative reverse transcriptase-mediated polymerase chain reaction

Total RNA was isolated from whole kidney using the guanidine–isothiocyanate based-reagent Isogen (Nippon Gene, Tokyo, Japan) according to the manufacturer’s instructions.

We performed real-time polymerase chain reaction analysis using probe sets from the Bio-Rad CFX96 system (Bio-Rad Laboratories Inc., Hercules, CA, USA). Gene-specific primers for Nrf2 (NM_031789.1; forward: GAGACGGCCATGACTGAT, reverse: GTGAGGGGATCGATGAGTAA) and glyceraldehyde-3-phosphate dehydrogenase (GAPDH, NM_017008.3; forward: GGCACAGTCAAGGCTGAGAATG, reverse: ATGGTGGTGAAGACGCCAGTA) were used for amplification of specific cDNAs with the iScript one step RT-PCR kit from Bio-Rad. The relative expression levels of each messenger RNA (mRNA) were normalized to GAPDH mRNA levels.

### Western blot analysis

For western blot analysis (10 μg protein per lane), frozen renal and cardiac tissues were homogenized and denatured by boiling in LDS sample buffer and sample reducing agents. Gels were then transferred to a PVDF membrane (Immobilon-P, Millipore Corporation, Billerica, MA, USA) using a semi-dry Transblot apparatus (Bio-Rad Laboratories Inc.). Membranes were blocked, probed with the specified antibodies, and then incubated with horseradish peroxidase-conjugated secondary antibodies prior to chemiluminescence detection (Pierce, Rockford, IL, USA). Band intensities were quantified by densitometry using Image J software. The antibodies used were: anti-Nrf2 (Abcam), anti-SOD2 rabbit polyclonal (ab13533) (Abcam), anti-gp91^phox^(C-15) (Santa Cruz Biotechnology Inc., Santa Cruz, CA, USA), and anti-p40^phox^ (N-20) (Santa Cruz Biotechnology Inc.).

### NADPH oxidase activity measurement

NADPH oxidase-dependent O_2_^−^ production by intact renal and cardiac tissue was measured using lucigenin-enhanced chemiluminescence. Briefly, 10 μl of the homogenate was transferred into glass scintillation vials containing 5 μmol/L lucigenin in Krebs-HEPES buffer (180 μl). The chemiluminescence value was recorded at 60 s intervals for 10 min and endpoint values were measured using a GENios pro luminescence reader (Tecan Co. Ltd., Kawasaki, Japan).

### Echocardiography

We performed transthoracic echocardiography with an echocardiographic system equipped with a 12-Mhz phased-array transducer (Aplio, Toshiba Medical Systems Corp., Otawara, Japan). Rats were anesthetized with 1% pentobarbital (0.20 mg/kg, IP) and left ventricular end-diastolic diameter (LVEDD), fractional shortening (FS), and left ventricle posterior wall thickness in diastole (LVPWTd) were measured using M-mode tracings.

### Statistical analyses

Data are expressed as mean ± standard error of mean, and were analyzed using the independent t-test or two-way repeated-measures ANOVA. Differences between the groups were considered significant at p < 0.05. All analyses were performed using Sigmastat 3.5 software (Systat Software, Chicago, IL, USA).

## Results

### Characterization of functional and histological changes in the kidney and heart of Dahl SS rats fed a normal salt diet and FW for 48 weeks

**Figure 1:**
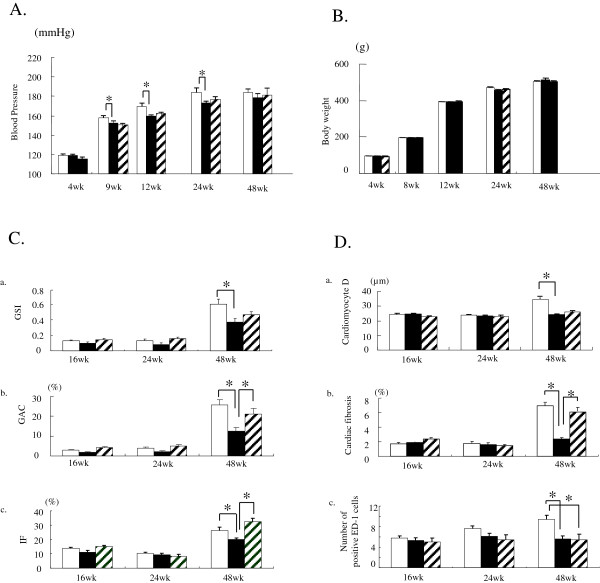
**Characterization of functional and histological changes in graphs. A** Changes in systolic blood pressure of rats fed FW □, EW ■, or DW ▨. **B** Changes in body weight of rats fed FW, EW, or DW. **C** Semi-quantitative analysis of histological changes in the kidneys of rats fed FW, EW, or DW. **a**, glomerular sclerosis index (GSI); **b**, glomerular adhesion and crescent (GAC); **c**, interstitial fibrosis (IF). *P < 0.05 versus 16 weeks. ^§^P < 0.0 g versus 24 weeks. **D** Semi-quantitative analysis of histological changes in the kidneys of rats fed FW, EW, or DW. **a**, cardiomyocyte diameter (cardiomyocyte D); **b**, cardiac fibrosis; **c**, ED-1, number of positive ED-1 (CD68) cells. *P < 0.05 versus 16 weeks. ^§^P < 0.0 g versus 24 weeks.

There were significant elevations in BP and BW in the FW group during the course of the study, which spanned a treatment duration of 48 weeks (Figure 1A, B).

### Kidney function in the FW group

During the course of the study, there were significant time-dependent increases in urinary albumin excretion in the FW group (p < 0.05 vs. 16 weeks) (Table 2). Results of semi-quantitative analysis of histological changes in the kidney are shown in Figure 1C. There were significant increases at 48 weeks compared to 16 and 24 weeks in the following parameters: glomerular sclerosis index (GSI; p < 0.05 vs. 16 and 24 weeks) (Figure 1C-a), glomerular adhesions and crescents (GAC; p < 0.05 vs. 16 and 24 weeks) (Figure 1C-b) and renal fibrosis (IF; p < 0.05 vs. 16 and 24 weeks) (Figure 1C-c).

**Table 2:** Biochemical parameters in the three groups

		**FW**			**EW**			**DW**	
	16^th^wk	24^th^wk	48^th^wk	16^th^wk	24^th^wk	48^th^wk	16^th^wk	24^th^wk	48^th^wk
Cr (mg/dl)	0.5 ± 0.0	0.7 ± 0.0	0.6 ± 0.0	0.5 ± 0.0	0.6 ± 0.0	0.6 ± 0.0	0.5 ± 0.0	0.6 ± 0.0	0.6 ± 0.0
BUN (mg/dl)	N/A	N/A	21.2 ± 0.5	N/A	N/A	21.8 ± 0.5	N/A	N/A	20.5 ± 0.4
UAE (mg/day)	5.9 ± 1.1	96.6 ± 20.0^*a*^	85.0 ± 22.1^*a*^	3.6 ± 0.7	110.7 ± 30.6^*a*^	197.2 ± 54.4^*a*^	4.0 ± 0.4	73.8 ± 11.9^*a*^	139.0 ± 24.5^*a*^
CCr (ml/min)	59.2 ± 7.0	39.0 ± 2.7	N/A	60.7 ± 10.0	44.2 ± 2.9	N/A	56.4 ± 8.8	45.2 ± 4.0	N/A

(mean±*SEM*).

*FW* filtered water, *EW* water with dissolved H_2_ produced by electrolysis, *DW* dehydrogenated EW. *Cr* creatinine,

*UAE* urinary albumin excretion, *BUN* blood urea nitrogen, *CCr* creatinine clearance, *BW* bodyweight, *N*/*A* not available.

^*a*^ P < 0.05, vs. 16^th^ wk.

### Heart

Echocardiographic observations in the FW group (at 48 weeks) are summarized in Table 3. There were significant increases in LVPWTd (p < 0.05 vs. 16 weeks) with aging. Analyses of the histological changes in the heart are shown in Figure 1D. There were significant increases at 48 weeks compared to 16 and 24 weeks in the following parameters: cardiomyocyte diameter (p < 0.05 vs. 16 and 24 weeks) (Figure 1D-a), cardiac fibrosis (p < 0.05 vs. 16 and 24 weeks) (Figure 1D-b), and number of ED-1 positive cells (p < 0.05 vs. 16 and 24 weeks) (Figure 1D-c).

**Table 3:** Serial changes in echocardiographic findings in the three groups

		**FW**			**EW**			**DW**	
	16^th^wk	24^th^wk	48^th^wk	16^th^wk	24^th^wk	48^th^wk	16^th^wk	24^th^wk	48^th^wk
FS (%)	29.7 ± 1.5	30.0 ± 1.0	32.1 ± 1.1	28.8 ± 0.6	29.2 ± 1.0	32.1 ± 1.1	28.6 ± 0.7	30.5 ± 0.6	31.8 ± 1.0
LVEDD (mm)	9.5 ± 0.1	9.3 ± 0.1	9.1 ± 0.1	9.3 ± 0.1	9.3 ± 0.1	9.6 ± 0.1	9.4 ± 0.1	9.5 ± 0.1	9.4 ± 0.1
LVPWTd (mm)	1.8 ± 0.1	1.7 ± 0.0	2.1 ± 0.0^*a*^	1.7 ± 0.0	1.7 ± 0.0	1.9 ± 0.0^*b*^	1.7 ± 0.0	1.6 ± 0.0	2.0 ± 0.0
E/A	1.6 ± 0.1	1.5 ± 0.1	1.4 ± 0.1	1.6 ± 0.1	1.3 ± 0.0	1.3 ± 0.1	1.5 ± 0.1	1.3 ± 0.1	1.3 ± 0.1

(mean±*SEM*).

*FW* filtered water, *EW* water with dissolved H_2_ produced by electrolysis, *DW* dehydrogenated EW. *FS* fractional shortening,

*LVEDD* left ventricular end-diastolic diameter, *LVPWTd* Left Ventricle Posterior Wall Thickness in Diastole, *BP* blood pressure.

^*a*^P < 0.05, vs. 16^th^ w, ^*b*^P < 0.05, EW vs. FW of 48^th^ week.

### Effect of H_2_-enriched electrolyzed water by ad libitum drinking

No differences were found between the three groups in terms of body weight (Figure 1B) and water and food consumption (data not shown) (FW vs. EW and DW). There were significant elevations in BP in the EW and DW groups at the four time points where BP was measured (8, 16, 24, and 48 weeks) in relation to baseline BP (at 4 weeks) (p < 0.05 vs. 4 weeks) (Figure 1A). Significant differences in BP were present at 16, 24, and 48 weeks between the FW and EW groups (p < 0.05 vs. FW) (Figure 1A). No differences were found in organ to body weight ratio analysis at 16, 24, and 48 weeks among the three groups (data not shown).

### Kidney

**Figure 2:**
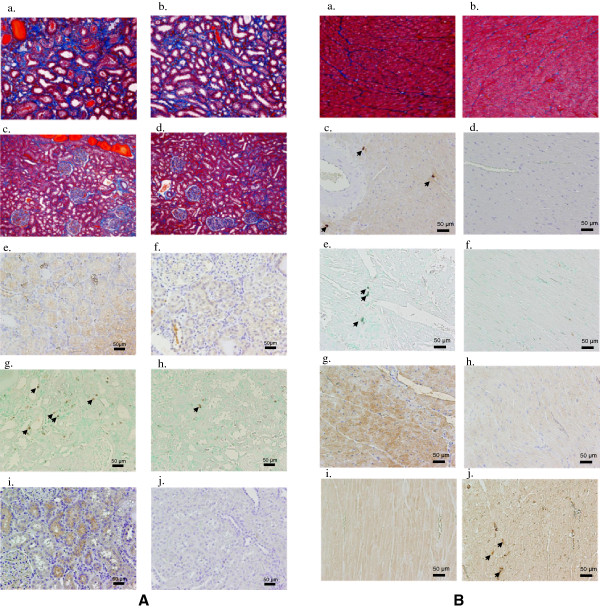
**Characterization of functional and histological changes in pictures. A** Representative histological findings in the kidneys of rats fed FW or EW for 43 weeks. Enhanced interstitial fibrosis in the outer renal medulla is observed in the FW **(a)** group compared to the EW **(b)** group. Exaggerated tubular dilatations are present in the FW **(c)** group as compared to the EW **(d)** group. Nitrotyrosine **(e)**, number of ED-1 positive cells (→) **(g)**, and malondialdehyde **(i)** were increased in the FW **(g, j, m)** group as compared to the EW **(f, h, i)** group in the renal cortex of rats. **B**. Representative histological findings in the hearts of rats fed FW or EW for 48 weeks. Enhanced cardiac fibrosis is found in the FW **(a)**, compared to the EW **(b)** group. Staining of nitrotyrosine positive cells (→) **(c)**, number of ED-1 positive cells (→) **(e)**, and malondialdehyde **(g)** were observed in the FW group, while faint staining was observed in the EW **(d, f, h)** group in the renal cortex of rats. Increased Nrf2 staining was observed in the EW **(j)** group as compared to the FW **(i)** group.

Kidney function analysis during the course of the study indicated that there were significant increases in urinary albumin excretions in the EW and DW groups (p < 0.05 vs. 16 weeks) (Table 2), although no differences were found in albumin excretion and serum creatinine levels compared to FW. Comparisons between FW and EW using semi-quantitative analysis of histological changes in the kidney are shown in Figure 1 C-D. There were significant increases at 48 weeks compared to 16 and 24 weeks in the following parameters in the EW and DW groups: GSI (Figure 1C-a), glomerular adhesions and crescents (Figure 1C-b), and renal fibrosis (Figure 1C-c). However, the changes at 48 weeks were significantly smaller in the EW group compared to the FW group (p < 0.05, respectively). In addition, there were significant differences in ED-1 positive cells (Figure 1D-c), and NT and MDA staining at 48 weeks, which were significantly less in the EW group compared to the FW group (p < 0.05, respectively). Representative histological findings in kidneys for each of the three groups at 48 weeks are shown in Figure 2A.

### Heart

Echocardiographic parameter for the EW and DW groups are shown in Table 3. There were significant increases in LVPWTd with aging in both groups (p < 0.05 vs. 16 and 24 weeks). However, LVPWTd of EW was significantly less than that of FW at 48 weeks (p < 0.05, EW vs. FW), indicating ameliorated cardiac remodeling in the EW group.

Analyses of histological changes in the heart are shown in Figure 1D. In all three groups, there were significant increases at 48 weeks compared to 16 and 24 weeks in the following parameters: cardiomyocyte diameter (p < 0.05 vs. 16 and 24 weeks) (Figure 1D-a), cardiac fibrosis (p < 0.05 vs. 16 and 24 weeks) (Figure 1D-b), and number of ED-1 positive cells (p < 0.05 vs. 16 and 24 weeks) (Figure 1D-c). However, the histological changes were significantly smaller in the EW group compared to the FW group (p < 0.05, EW vs. FW) at 48 weeks. In addition, NT and MDA staining at 48 weeks were significantly less in the EW group compared to the FW group (p < 0.05, respectively) (Table 4).

**Table 4:** Comparison of oxidant and anti-oxidant systems between FW and EW groups at 48 wks

	**FW**	**EW**
Kidney		
Nitrotyrosine (positive area%)	6.3 ± 0.7	4.3 ± 0.3^a^
Malondialdehyde (positive area%)	60.5 ± 1.2	57.2 ± 1.2^a^
ED1	113.5 ± 13.4	75.3 ± 8.2^a^
Heart		
Nrf2 (Nrf2/beta actin)	1.0 ± 0.4	2.4 ± 0.6^a^
Number of Nrf2 (per slide)	1.9 ± 0.4	10.7 ± 1.4^a^
gp91phox (gp91phox/beta actin)	7.5 ± 2.4	1.1 ± 0.4^a^
Nitrotyrosine (positive area%)	49.8 ± 6.0	37.8 ± 6.7^a^
Malondialdehyde (positive area%)	63.7 ± 2.6	44.6 ± 1.2

(mean±SEM).

*FW* filtered water, *EW* water with dissolved H_2_ produced by electrolysis, *DW* dehydrogenated EW.

Nrf2, nuclear factor (erythroid-derived 2)-like 2.

*NADPH* oxidase activity, nicotinamide adenine dinucleotide phosphate-oxidase.

gp91phox and p40phox, phagocytic oxidase unit of *NADPH* oxidase.

*SOD*2, Superoxide dismutase 2.

^*a*^ P < 0.05, EW vs. *FW*.

Representative histological findings in the hearts of the animals in the three groups at 48 weeks are shown in Figure 2b.

### Possible mechanism of cardio-renal protection by H_2_-enriched water (Additional file 1: Table S1)

In the kidney, no differences were found in the expression of Nrf2 and NADPH oxidase activity at 48 weeks (Additional file 1: Table S1). In the heart, although no differences were found in Nrf2 mRNA levels among the groups, immunostaining and western blot analysis showed significantly higher levels in the EW compared to the FW group (p < 0.05) (Table 4). No statistical differences were found in NADPH oxidase activity among groups, although activity in the EW group tended to be lower than in the FW group, and significantly lower expression of the gp91phox subunit in the EW group compared to the FW group (Table 4).

## Discussion

In the present study, Dahl SS male rats were fed a 0.5% salt diet and age-related changes were observed for up to 48 weeks. Although no premature deaths were found during the study period, there were progressive elevations in BP, with accompanying increases in albuminuria and cardiac remodeling. Histologically, there was pronounced glomerular sclerosis with tubulointerstitial fibrosis in the kidney, and increased cardiomyocyte diameter with interstitial fibrosis in the heart at 48 weeks. These changes, as a function of aging, were related to enhanced inflammation (infiltration of ED-1 positive cells) and oxidative stress (MDA, NT staining) in the respective organs.

We examined the potential of employed H_2_-enriched EW as an anti-oxidant to suppress organ damage in rats. They were allowed *ad libitum* drinking of H_2_-enriched EW, which was replaced twice a day to ensure consistent H_2_ content in the water (492.5 ppb). Interestingly, despite the fact that there were no striking differences in BP between the FW and EW groups, there were fewer histological alterations in the EW group with concurrently lower levels of oxidative stress and inflammatory markers. Thus, it is surmised that enhancement of oxidative stress could, at least partially, play a role in organ injury, independent of BP elevation. It is thought that long-term H_2_ administration could enable the amelioration of the oxidative stress-dependent pathological processes in our model.

Recently, a series of studies revealed that H_2_ exerts anti-oxidative and anti-inflammatory effects. Pre-treatment by inhalation of H_2_ gas or loading with H_2_ rich water, generated by H_2_ bubbling or electrolysis, have demonstrated biological effects in organs, resulting in protection against ischemia and inflammatory injuries [1,3,5-7,10,12,14,15,20]. It has been proposed that H_2_ reacts with super-oxide anions to deliver H_2_O and atomic H, which quenches various types of radicals [12]. Recent cumulative evidences have revealed that H_2_ administration leads to decreases in oxidative stress markers, pro-inflammatory molecules, and apoptosis, likely involving mechanisms such as the suppression of signaling pathways for MAPK, MEK-1, and caspase, while increasing anti-oxidant molecules [1,5-7,14,15,20].

Interestingly, the present study found a significant increase in Nrf2 protein expression in the heart of EW rats, suggesting the up-regulation of anti-oxidative stress mechanisms, while the expression of NADPH oxidase subunits was suppressed in the heart. Analyzing our results and those of previous reports, we can speculate that EW exhibits anti-oxidative and anti-inflammatory actions by multiple independent mechanisms, such as suppression of super-oxide production by modulation of NADPH oxidase, and activation of a key player in the anti-oxidant cell defense system, such as Nrf2 expression.

Oxidative stress is thought to play a crucial role in aging, and organs with limited cell proliferation, i.e., the kidney and heart tend to show an accumulation of DNA damage with age [9]. Therefore, it is possible that long-term H_2_ administration may have ameliorated the pathological process of aging in rats in the present study. The effects of H_2_ need to be addressed in terms of cell senescence in the future [18].

The NADPH oxidase system and superoxide were recently implicated in cardio-renal injury [21], while SOD2, one of the Nrf2 target genes, was moderately elevated in the EW group (Additional file 1: Table S1).

In conclusion, the results of the present study showed that long-term *ad libitum* consumption of H_2_-enriched water could ameliorate age-related cardio-renal injury in Dahl SS rats on a normal salt diet. This suggests the potential of utilizing H_2_ water as a novel anti-aging strategy.

## Competing interests

The authors declare that they have no competing interests.

## Authors’ contributions

W-JZ: Practioner, planning, project, protocols, analysis, writer. MN: Planning, project, writer, adviser. TM: Adviser; KH: Practioner; HT: Adviser; JK: Adviser; SK: Adviser; SI: Adviser. All authors read and approve the final manuscript.

## Supplementary Material

Additional file 1: Table S1 Comparison of oxidant and anti-oxidant systems between FW and EW groups at 48 wks. Click here for file

## References

[B1] Buchholz BM Kaczorowski DJ Sugimoto R Yang R Wang Y Billiar TR McCurry KR Bauer AJ Nakao A Hydrogen inhalation ameliorates oxidative stress in transplantation induced intestinal graft injury Am J Transplant 2008 8 10 2015 2024 18727697 10.1111/j.1600-6143.2008.02359.x

[B2] Forde P Scribner AW Dial R Loscalzo J Trolliet MR Prevention of hypertension and renal dysfunction in Dahl rats by alpha-tocopherol J Cardiovasc Pharmacol 2003 42 1 82 88 12827031 10.1097/00005344-200307000-00013

[B3] Fukuda K Asoh S Ishikawa M Yamamoto Y Ohsawa I Ohta S Inhalation of hydrogen gas suppresses hepatic injury caused by ischemia/reperfusion through reducing oxidative stress Biochem Biophys Res Commun 2007 28;361 3 670 674 10.1016/j.bbrc.2007.07.088 17673169

[B4] Kim MJ Kim HK Anti-diabetic effects of electrolyzed reduced water in streptozotocin-induced and genetic diabetic mice Life Sci 2006 10;79 24 2288 2292 10.1016/j.lfs.2006.07.027 16945392

[B5] Kim MJ Jung KH Uhm YK Leem KH Kim HK Preservative effect of electrolyzed reduced water on pancreatic beta-cell mass in diabetic db/db mice Biol Pharm Bull 2007 30 2 234 236 17268057 10.1248/bpb.30.234

[B6] Lee MY Kim YK Ryoo KK Lee YB Park EJ Electrolyzed-reduced water protects against oxidative damage to DNA, RNA, and protein Appl Biochem Biotechnol 2006 135 2 133 144 17159237 10.1385/abab:135:2:133

[B7] Liu S Liu K Sun Q Liu W Xu W Denoble P Tao H Sun X Consumption of hydrogen water reduces paraquat-induced acute lung injury in rats J Biomed Biotechnol 2011 2011 305086 21318114 10.1155/2011/305086 PMC3035012

[B8] Mikoda N Nakagawa H Kawahara K Nihon Yakurigaku Z Effects of nitrendipine on the development of hypertension and renal failure in Dahl salt-sensitive rats [in Japanese] Magazine 1999 114 6 373 382 10.1254/fpj.114.373 10672598

[B9] Møller P Løhr M Folkmann JK Mikkelsen L Loft S Aging and oxidatively damaged nuclear DNA in animal organs Free Radic Biol Med 2010 48 10 1275 1285 20149865 10.1016/j.freeradbiomed.2010.02.003

[B10] Nagata K Nakashima-Kamimura N Mikami T Ohsawa I Ohta S Consumption of molecular hydrogen prevents the stress-induced impairments in hippocampus-dependent learning tasks during chronic physical restraint in mice Neuropsychopharmacology 2009 34 2 501 508 18563058 10.1038/npp.2008.95

[B11] Nishiyama A Yoshizumi M Hitomi H Kagami S Kondo S Miyatake A Fukunaga M Tamaki T Kiyomoto H Kohno M Shokoji T Kimura S Abe Y The SOD mimetic tempol ameliorates glomerular injury and reduces mitogen-activated protein kinase activity in Dahl salt-sensitive rats J Am Soc Nephrol 2004 15 2 306 315 14747377 10.1097/01.asn.0000108523.02100.e0

[B12] Ohsawa I Ishikawa M Takahashi K Watanabe M Nishimaki K Yamagata K Katsura K Katayama Y Asoh S Ohta S Hydrogen acts as a therapeutic antioxidant by selectively reducing cytotoxic oxygen radicals Nat Med 2007 13 6 688 694 17486089 10.1038/nm1577

[B13] Quaschning T D’Uscio LV Shaw S Gröne HJ Ruschitzka F Lüscher TF Vasopeptidase inhibition restores renovascular endothelial dysfunction in salt-induced hypertension J Am Soc Nephro 2001 12 11 2280 2287 10.1681/ASN.V12112280 11675404

[B14] Shirahata S Kabayama S Nakano M Miura T Kusumoto K Gotoh M Hayashi H Otsubo K Morisawa S Katakura Y Electrolyzed-reduced water scavenges active oxygen species and protects DNA from oxidative damage Biochem Biophys Res Commun 1997 234 1 269 274 9169001 10.1006/bbrc.1997.6622

[B15] Spulber S Edoff K Hong L Morisawa S Shirahata S Ceccatelli S Molecular hydrogen reduces LPS-induced neuroinflammation and promotes recovery from sickness behaviour in mice PLoS One 2012 7 7 e42078 Epub 2012 Jul 31 22860058 10.1371/journal.pone.0042078 PMC3409143

[B16] Tian N Thrasher KD Gundy PD Hughson MD Manning RD Jr Antioxidant treatment prevents renal damage and dysfunction and reduces arterial pressure in salt-sensitive hypertension Hypertension 2005 45 5 934 939 15837840 10.1161/01.HYP.0000160404.08866.5a

[B17] Tian N Moore RS Braddy S Rose RA Gu JW Hughson MD Manning RD Jr Interactions between oxidative stress and inflammation in salt-sensitive hypertension Am J Physiol Heart Circ Physiol 2007 293 6 H3388 H3395 Epub 2007 Oct 5 17921322 10.1152/ajpheart.00981.2007

[B18] Yang H Fogo AB Cell senescence in the aging kidney J Am Soc Nephrol 2010 21 9 1436 1439 20705707 10.1681/ASN.2010020205

[B19] Zhang L Fujii S Igarashi J Kosaka H Effects of thiol antioxidant on reduced nicotinamide adenine dinucleotide phosphate oxidase in hypertensive Dahl salt-sensitive rats Free Radic Biol Med 2004 37 11 1813 1820 15528040 10.1016/j.freeradbiomed.2004.08.019

[B20] Zhu WJ Nakayama M Mori T Nakayama K Katoh J Murata Y Sato T Kabayama S Ito S Intake of water with high levels of dissolved hydrogen (H_2_) suppresses ischemia-induced cardio-renal injury in Dahl salt-sensitive rats Nephrol Dial Transplant 2011 26 7 2112 2118 21193644 10.1093/ndt/gfq727

[B21] Aroor AR Mandavia C Ren J Sowers JR Pulakat L Mitochondria and oxidative stress in the cardiorenal metabolic syndrome Cardiorenal Med 2012 2 2 87 109 22619657 10.1159/000335675 PMC3357146

