# Supplementary material for: Amelioration of cardio-renal injury with aging in dahl salt-sensitive rats by H2-enriched electrolyzed water
Source: Med Gas Res. 2013 Dec 2;3:26. doi: 10.1186/2045-9912-3-26 (PMC3866609; doi:10.1186/2045-9912-3-26)
Supplement: Additional file 1: Table S1 — Comparison of oxidant and anti-oxidant systems between FW and EW groups at 48 wks. [file 2045-9912-3-26-S1.docx]

Table(supplement). Comparison of oxidant and anti-oxidant systems between FW and EW groups at 48 wks

|  | FW | EW |
| --- | --- | --- |
| Kidney |  |  |
| Nrf2 (Nrf2/GAPDH) | 3.7±0.5 | 4.2±0.4 |
| Nrf2 (Nrf2/beta actin) | 0.8±0.2 | 0.6±0.2 |
| NADPH oxidase activity (cpm/ug protein) | 2344.5±279.8 | 1661.6±351.7 |
| Heart |  |  |
| Nrf2 (Nrf2/GAPDH) | 1.0±0.0 | 0.8±0.0 |
| p40phox (p40phox/beta actin) | 2.2±0.8 | 1.21±0.5 |
| NADPH oxidase activity (cpm/ug protein) | 1028.5±394.5 | 491.9±124.0 |
| SOD2 (SOD2/beta actin) | 2.9±0.4 | 4.4±0.4 |

(mean±SEM)

FW, filtered water; EW, water with dissolved H_2_ produced by electrolysis; DW, dehydrogenated EW.

Nrf2, nuclear factor (erythroid-derived 2)-like 2;

NADPH oxidase activity, nicotinamide adenine dinucleotide phosphate-oxidase,

gp91phox and p40phox, [phagocytic](http://ja.wikipedia.org/wiki/%E9%A3%9F%E4%BD%9C%E7%94%A8) [oxidase](http://ja.wikipedia.org/wiki/%E9%85%B8%E5%8C%96%E9%85%B5%E7%B4%A0) unit of NADPH oxidase,

SOD2, Superoxide dismutase 2.

*^a^* P<0.05, EW vs. FW.
